# Celluloid Manufacturing Company

**Published:** 1878-02

**Authors:** C. Stoddard Smith


### CELLULOID MANUFACTURING COMPANY.

To the Editor of the Dental Register:—The Celluloid Manufacturing Company, now that the suits in regard to the infringement of the Cummings patent have been definitely settled in their favor, thus securing to dentists the right to use the celluloid base without molestation, have determined to devote more attention to the dental department of their business. They have engaged the services of the undersigned, who will make it his busines, to answer all inquiries, as well as to improve the celluloid base in its application to dentistry. They will be pleased to receive suggestions from any quarter, and to reply to any questions that may be asked. Every suggestion that appears to be valuable will be put to a practical test, and such as prove so, will be made use of.

Several styles of plates not generally in use, are now for sale, and other forms will soon be prepared. Attention is called at present to the following as not generally known. "A" plates, shapes and sizes the same as the ordinary uppers, but nearly double in thickness, for cases where much absorption has taken place; "plumper" plates, four sizes (uppers) with very heavy rim, as indicated by the name, and a new style of partial plates, (two sizes Nos. 7 and 8) for cases where only the front teeth are to be replaced. These are all very useful plates, and should be in the hands of every dentist who uses celluloid in working which it is important to have a plate of the proper size and shape at hand.

If dentists can not obtain these plates from the depots, orders sent directly to the factory will be filled through Dr. Sam'l S. White, sole agent for the sale of the same.

A further improvement in the forms of plates is contemplated, due notice of which will be given. There is also in preparation a small pamphlet, containing revised instructions, testimonials, etc., and descriptions and cuts of the plates, a copy of which when issued, will be furnished on application.

The superiority of celluloid over rubber in many if not all respects, is now acknowledged by those who have made the most extensive use of it. The Celluloid Manufacturing Company is in possession of testimonials from those who have used it to the extent of hundreds of plates, during periods extending over five or six years, certifying to its being more satisfactory to both, dentist and patient. It is less heating to the gums, free from injurious ingredients, and far more pleasant and cleanly, both to manufacture and wear besides being infinitely preferable in appearance. The use of plain teeth enables the dentist to give a far more life-like and natural expression to the features, than with gum sections.

The undersigned may be addressed as above, and will be pleased to answer any inquiries.

C. STODDARD SMITH.

